# Enhancing prognosis prediction using pre-treatment nodal SUVmax and HPV status in cervical squamous cell carcinoma

**DOI:** 10.1186/s40644-019-0226-4

**Published:** 2019-06-24

**Authors:** Chae Moon Hong, Shin-Hyung Park, Gun Oh Chong, Yoon Hee Lee, Ju Hye Jeong, Sang-Woo Lee, Jaetae Lee, Byeong-Cheol Ahn, Shin Young Jeong

**Affiliations:** 1 Department of Nuclear Medicine, School of Medicine, Kyungpook National University, Daegu, Republic of Korea; 2 Department of Obstetrics and Gynecology, School of Medicine, Kyungpook National University, Daegu, Republic of Korea; 3 Department of Radiation Oncology, School of Medicine, Kyungpook National University, Daegu, Republic of Korea; 4 Department of Nuclear Medicine, Kyungpook National University Hospital, 130 Dongdeok-ro, Jung-gu, Daegu, 41944 Republic of Korea; 5 Department of Nuclear Medicine, School of Medicine, Kyungpook National University Chilgok Hospital, 807, Hoguk-ro, Buk-gu, Daegu, 41404 Republic of Korea; 6 Department of Obstetrics and Gynecology, School of Medicine, Kyungpook National University Chilgok Hospital, 807, Hoguk-ro, Buk-gu, Daegu, 41404 Republic of Korea; 7 Department of Radiation Oncology, Kyungpook National University Chilgok Hospital, 807 Hoguk-ro, Buk-gu, Daegu, 41404 Republic of Korea

**Keywords:** Cervical cancer, FDG PET/CT, Lymph node, Human papilloma virus, Prognosis prediction, Classification and regression tree, Concurrent chemoradiotherapy

## Abstract

**Background:**

This study was to evaluate the prognostic value of metabolic parameters on F-18-FDG PET/CT and the status of human papillomavirus (HPV) infection and known prognostic variables for predicting tumor recurrence and investigating a prognostic model in patients with locally advanced cervical cancer treated with concurrent chemoradiotherapy (CCRT).

**Methods:**

A total of 129 patients with cervical squamous cell carcinoma who underwent initial CCRT were eligible for this study. Univariate and multivariate analyses were performed using traditional prognostic factors, metabolic parameters, and HPV infection. Classification and regression decision tree (CART) was used to establish new classification.

**Results:**

Among 129 patients, 29 patients (22.5%) had recurrence after a median follow-up of 60 months (range, 3–125 months). Tumor size, para-aortic lymph node metastasis, nodal SUVmax, and HPV infection status were identified as independent prognostic factors by multivariate analysis. The CART analysis classified the patients into three groups. The first node was nodal SUVmax, and HPV status was the second node for patients with nodal SUVmax ≤7.49; Group A (nodal SUVmax ≤7.49 and HPV positive, HR 1.0), Group B (nodal SUVmax ≤7.49 and HPV negative, HR 3.56), and Group C (nodal SUVmax > 7.49, HR 10.13). Disease-free survival was significantly different among the three groups (*p* < 0.001).

**Conclusion:**

The nodal SUVmax on F-18 FDG PET/CT and HPV infection status before CCRT are powerful independent prognostic factors for the prediction of disease-free survival in patients with cervical squamous cell carcinoma who underwent initial CCRT. We also suggest a simple prognosis prediction model using pre-treatment FDG PET/CT and HPV genotyping; however, it needs further validation in an independent dataset.

**Electronic supplementary material:**

The online version of this article (10.1186/s40644-019-0226-4) contains supplementary material, which is available to authorized users.

## Background

Cervical cancer is a commonly diagnosed cancer and the fourth leading cause of cancer-related deaths in women worldwide [1]. Surgery and cisplatin-based concurrent chemoradiotherapy (CCRT) are the primary treatment options for cervical cancer. However, about one third of patients with cervical cancer experience recurrence, and recurrence mostly develops within 2 years of therapy completion [2, 3]. Therefore, accurate prognosis prediction is important to improve the treatment decision and survival of patients.

Advanced International Federation of Gynecology and Obstetrics (FIGO) stage, age of patients, tumor histology, size of primary tumor, and the pelvic/para-aortic lymph node status are the traditional prognostic factors for patients with cervical cancer [3, 4]. However, these factors are not enough for predicting recurrence, and there is continuous dispute about these factors except lymph node metastasis [3, 5]. Factors for the current FIGO staging system do not include lymph node metastasis, but the status of lymph node metastasis is well known as a powerful prognostic factor [4, 6].

Although there is much evidence supporting the role of human papillomaviruses (HPVs) in the development of cervical precursor lesions and invasive cervical cancer, the prognostic value of HPV deoxyribonucleic acid (DNA) is not well understood. Moreover, the existing results on the relationship between HPV genotype and survival are heterogeneous. The etiology of squamous cell carcinoma is firmly linked to infection with HPVs [7] and to the prognosis of the patients [8, 9]. Some studies reported poorer prognosis with specific genotypes of HPV, such as HPV18 or alpha-7 strain, and multiple infections of different genotypes of HPV than with single genotype involvement [10–13]. However, other reports did not uphold the prognostic value of HPV 18 and alpha-7 strain [8, 14], and mixed infection of alpha-7 and alpha-9 strains showed modest prognosis [9]. A recent meta-analysis demonstrated that HPV-positive DNA status before treatment was associated with a favorable prognosis in patients with cervical cancer [15]. Moreover, our recent studies showed that HPV negativity was associated with worse survival outcomes [8, 9].

Metabolic parameters from F-18 fluorodeoxyglucose (FDG) positron emission tomography/computed tomography (PET/CT) are widely studied and showed promising results for predicting recurrence [3, 6, 16]. Metabolic parameters from F-18 FDG PET/CT can be obtained without surgery or invasive procedures; thus, prognosis assessment using F-18 FDG PET/CT is more suitable for patients who are eligible for CCRT. Nevertheless, to our knowledge, a risk stratification model using metabolic variables on PET/CT combined with other known prognostic factors has not been proposed.

The purpose of this study was to evaluate the prognostic values of multiple parameters including traditional factors, glucose metabolism from F-18 FDG PET/CT, and HPV infection status for disease-free survival (DFS), and to investigate a prognosis prediction model using significant parameters in patients with cervical squamous cell carcinoma who underwent CCRT.

## Methods

### Patients

In this study, we enrolled cervical cancer patients with biopsy-confirmed squamous cell carcinoma of the cervix between August 2005 and June 2015, and we selected patients who underwent CCRT as their initial treatment. Among these patients, this study included the patients who underwent initial staging F-18 FDG PET/CT and HPV genotyping from cervico-vaginal swab before the initiation of CCRT. Clinical and pathologic parameters were obtained by retrospective chart review. Retrospective data collection and analysis were approved by the Institutional Review Board. The requirement for informed consent was waived due to the retrospective design of the study. The patients were staged according to the FIGO staging system. Patients who exhibited signs of distant metastatic disease or had a history of undergoing surgery, radiotherapy, or chemotherapy were excluded from the study. The clinical and pathological parameters were reviewed and retrieved, including age, serum squamous cell carcinoma antigen, FIGO stage, histology, primary tumor size, and the presence of pelvic and para-aortic lymph node metastasis.

### HPV genotyping

HPV genotyping was performed using cervico-vaginal swab specimens with the PANArray HPV Genotyping Chip (PANArray; PANAGENE, Daejeon, Korea) and the Anyplex II HPV 28 assay kit (Seegene, Seoul, Korea).

For the PANArray HPV Genotyping Chip, DNA was extracted using a heating method according to the manufacturer’s instructions. Briefly, 1 mL of a liquid-based preparation from the cervical specimen was transferred to phosphate-buffered saline. This mixture was centrifuged, and then 20 μL of DNA extraction buffer was added. The sample was heated at 55 °C for 1 h and then at 110 °C for 28 min. After centrifugation at 12,000 rpm for 5 min, 90 μL of the supernatant was used as the DNA template for polymerase chain reaction (PCR) analysis. With this assay, 32 HPV types could be simultaneously detected, including 20 high-risk and probable high-risk types (HPV 16, 18, 26, 31, 33, 34, 35, 39, 45, 51, 52, 53, 56, 58, 59, 66, 68, 69, 70, and 73) and 12 low-risk HPV types (HPV 6, 11, 32, 40, 42, 43, 44, 54, 55, 62,81, and 83).

The Anyplex II HPV 28 assay was performed according to the manufacturer’s instructions. Briefly, 5 μL of DNA was used in both 20-μL reactions, one with primer set A and the other with B. The Anyplex II HPV 28 assay uses HPV-specific dual priming oligonucleotides for multiplex (real-time) PCR. A total of 28 HPV types could be simultaneously detected, including 18 high-risk types (HPV 16, 18, 26, 31, 33, 35, 39, 45, 51, 52, 56, 58, 59, 66, 68, 69, 73, and 82) and 8 low-risk types (HPV 6, 11, 40, 42, 44, 53, 54, and 70).

### F-18 FDG pet/CT

Every patient underwent pre-treatment F-18 FDG PET/CT for initial staging work-up. All patients fasted for at least 6 h before undergoing the procedure, and their blood glucose levels were determined before the administration of F-18 FDG. Patients with blood glucose levels higher than 150 mg/dL were rescheduled for a later examination, and treatment was administered to maintain a blood glucose concentration of < 150 mg/dL in all subjects. Patients received intravenous injections of approximately 4.7 MBq of FDG per kg of body weight and were advised to rest for 1 h before F-18 FDG PET/CT image acquisition. F-18 FDG PET/CT scans were performed using a Reveal RT-HiREZ 6-slice CT apparatus (CTI Molecular Imaging, Knoxville, TN, USA) and a 16-slice CT Discovery STE apparatus (GE Healthcare, Milwaukee, WI, USA).

Before the PET scan, for attenuation correction, a low-dose CT scan without contrast enhancement was obtained from the skull base to the thigh when the patient was breathing quietly in supine position. PET scans with a maximum spatial resolution of 6.5 mm (Reveal PET/CT) and 5.5 mm (Discovery PET/CT) were also obtained from the skull base to the thigh at 3 min per bed for each patient; the maximum standardized uptake (SUVmax) was designated as the highest SUVmax of the primary tumor (pSUVmax) and lymph nodes (nodal SUVmax) on Advantage Workstation 4.3 (GE Healthcare, Milwaukee, WI, USA).

### Treatment and clinical follow-ups

All patients were treated with a combination of external beam radiotherapy and high-dose-rate intracavitary brachytherapy with curative intent. Radiotherapy comprised external beam radiotherapy (EBRT) and high-dose-rate intracavitary brachytherapy. EBRT was administered to the whole pelvis five times a week using 10-MV X-ray in 1.8 Gy daily fractions until a total dose of 45 Gy was reached. A four-field box technique was used. The superior border was the L4–L5 vertebral level. The inferior border was at the bottom of the obturator foramen or 2–3 cm below the lowest extent of the cervical or vaginal disease. The lateral borders were placed 2 cm laterally to the inner bony margins of the true pelvis. For the lateral fields, the anterior border included the symphysis pubis and the posterior border was the S2–3 interspace. For patients with para-aortic lymph node involvement, the extended pelvic field including para-aortic lymph nodes was used. A parametrial external beam boost of 10 Gy in five fractions was indicated in patients with parametrial involvement. Intracavitary brachytherapy was initiated after delivering an EBRT dose of 39.6 Gy. Intracavitary brachytherapy was delivered twice a week in five fractions, with a fractional dose of 6 Gy. Six cycles of weekly cisplatin were administered during radiotherapy at a dose of 40 mg/m^2^. The first course of cisplatin was administered on day 1 of radiotherapy.

Clinical follow-ups of patients were performed every 3 months until 2 years, every 6 months after 2 years, and annually thereafter. Tumor recurrence was defined as pathologically proven presence or progression of the disease on serial imaging studies. To evaluate the prognostic value of the clinical and metabolic parameters, DFS was chosen as an endpoint. DFS was calculated from the date of diagnosis of the disease to the date of diagnosis of recurrence or the date of the last follow-up.

### Statistical analysis

The data were analyzed using the R statistics (version 3.4.4), and *p* < 0.05 was considered to indicate statistical significance. Pearson’s chi-square test was used to evaluate the association between the variables. T-test or Wilcoxon rank-sum test was used for comparing continuous variables. The Cox-proportional hazard regression was performed analyzing DFS. Multivariate analysis was also performed using Cox-proportional hazard regression with backward elimination. Hazard ratio (HR) with 95% confidence intervals (CI) was provided for the model.

For further analysis of classification and regression tree (CART), variables with *p* < 0.1 in univariate analysis were considered as input variables: FIGO stage, size of primary tumor, pSUVmax, nodal SUVmax, HPV status, and the status of para-aortic lymph node metastasis. These factors were analyzed together using “ctree” of the “party” package. The input data of the CART analysis were the variables, time duration, and the status of DFS. Then, the tree chart was generated. The survival differences among the CART nodes were further verified using the log–rank test and Cox-proportional hazard regression, and survival graph was generated using the Kaplan–Meier method.

## Results

### Clinical features and treatment outcomes

One hundred and twenty-nine patients were identified in this study. The clinical characteristics of the study participants are listed in Table 1. The mean age was 54.0 ± 12.6 years. The most common FIGO stage was II (*n* = 76, 58.9%), followed by IV (*n* = 19, 14.7%), I (*n* = 17, 13.2%), and III (*n* = 17, 13.2%). The mean tumor size was 4.5 ± 1.5 cm, and mean serum squamous cell carcinoma (SCC) antigen level was 14.8 ± 28.1 ng/mL. Sixty-three patients (48.8%) had pelvic and/or para-aortic nodal metastases. Sixty-one patients (47.3%) showed pelvic lymph node metastasis, and 18 patients (14.0%) showed para-aortic lymph node metastasis. After a median follow-up of 60 months (range, 3–125 months), 29 patients (22.5%) had recurrence. Fifteen patients (51.7%) showed recurrence within the radiation field, 11 patients (37.9%) showed recurrence outside the radiation field, and three patients (10.3%) showed recurrence both inside and outside.

**Table 1:** Patient characteristics

Variables	*n* = 129	
Age	54.0 ± 12.6	
FIGO stage, n, (%)		
I	17	(13.2%)
II	76	(58.9%)
III	17	(13.2%)
IV	19	(14.7%)
Tumor size (cm)	4.5 ± 1.5	
SCC antigen (ng/mL)	14.8 ± 28.1	
Lymph node metastasis status, n, (%)		
Pelvic lymph node	61	(47.3%)
Para-aortic lymph node	18	(14.0%)
PET parameter		
pSUVmax	13.0 ± 6.9	
Nodal SUVmax	2.3 ± 5.0	
HPV infection, n (%)	111	(86.0%)
Median follow-up (months)	60	

*FIGO* International Federation of Gynecology and Obstetrics, *SCC* squamous cell carcinoma, *PET* positron emitting tomography, *SUVmax* maximum standardized uptake, *pSUVmax* SUVmax of primary tumor, *nodal SUVmax* SUVmax of the lymph node with the highest FDG uptake, *HPV* human papilloma virus

### Prevalence of HPV DNA genotype

Among 129 patients, 111 patients (86.0%) had HPV infection. The three most common HPV types were 16 (*n* = 72, 55.8%), 18 (*n* = 9, 7.0%), and 33 (*n* = 8, 6.2%). Multiple HPV infections were detected in 14 patients (10.9%). Further, 96 patients (74.4%) had HPVs of alpha-9 (HPV 16, 33, 58, 31, 35, and 52), and 10 patients (7.7%) had HPVs of alpha-7 (HPV 18, 39, 45, and 68). Three patients (2.3%) showed mixed infection of alpha-7 and alpha-9, and two patients (1.6%) showed other types of infection.

### PET parameters

The mean values of pSUVmax and nSUVmax were 13.0 ± 6.9 and 2.3 ± 5.0. Patients with recurrence showed higher pSUVmax than patients without recurrence (14.6 ± 4.9 vs. 12.6 ± 7.3, *p* = 0.005). The former also showed higher nSUVmax than the latter (5.1 ± 2.7 vs. 1.5 ± 2.7, *p* = 0.003).

### Univariate and multivariate analyses of DFS

In univariate analysis, the differences in FIGO stage (HR, 2.02; CI, 1.36–3.00; *p* < 0.001), tumor size (HR, 1.43; CI, 1.14–1.08; *p* = 0.002), the status of para-aortic lymph node metastasis (HR, 4.21; CI, 1.89–9.40; *p* < 0.001), nodal SUVmax (HR, 1.07; CI, 1.04–1.11; *p* < 0.001), and HPV negativity (HR, 2.37; CI, 1.01–5.55; *p* = 0.047) were statistically significant in DFS. However, there were no significant differences in terms of age, the status of pelvic lymph node metastasis, SCC antigen, and pSUVmax. In multivariate analysis, the differences in tumor size (HR, 1.37; CI, 1.06–1.77; *p* = 0.015), para-aortic lymph node metastasis (HR, 3.75; CI, 1.39–10.15; *p* = 0.0092), nodal SUVmax (HR, 1.05; CI, 1.01–1.09; *p* = 0.020), and HPV negativity (HR, 3.68; CI, 1.44–9.41; *p* = 0.007) were statistically significant. Results are summarized in Table 2.

**Table 2:** Univariate and multivariate analyses of disease-free survival

Variables	Univariate analysis			Multivariate analysis		
	HR	95% CI	*p*-value	HR	95% CI	*p*-value
Age^a^	0.98	0.95–1.01	0.140			
FIGO stage	2.02	1.36–3.00	< 0.001			
Tumor size^a^	1.43	1.14–1.08	0.002	1.37	1.06–1.77	0.015
Pelvic lymph node	1.89	0.90–3.96	0.094			
Para-aortic lymph node	4.21	1.89–9.40	< 0.001	3.75	1.39–10.15	0.009
SCC antigen^a^	1.01	1.00–1.01	0.122			
pSUVmax^a^	1.04	0.99–1.08	0.086			
Nodal SUVmax^a^	1.07	1.04–1.11	< 0.001	1.05	1.01–1.09	0.020
HPV negative	2.37	1.01–5.55	0.047	3.68	1.44–9.41	0.007

^a^Age, Tumor size, SCC antigen, pSUVmax, nodal SUVmax were analyzed as continuous variables. Hazard ratio represents the increase in hazard for each variable

*HR* hazard ratio, *FIGO* International Federation of Gynecology and Obstetrics, *SCC* squamous cell carcinoma, *SUVmax* maximum standardized uptake, *pSUVmax* SUVmax of primary tumor, *nodal SUVmax* SUVmax of the lymph node with the highest FDG uptake, *HPV* human papilloma virus

As the CART analysis provided cut-off values of continuous variables, we performed additional univariate and multivariate analyses. However, a cut-off value could not be obtained for age, SCC, and pSUVmax due to their statistically less significant predictive power for DFS (Additional file 1: Table S1).

### Classification and regression tree

**Fig. 1:**
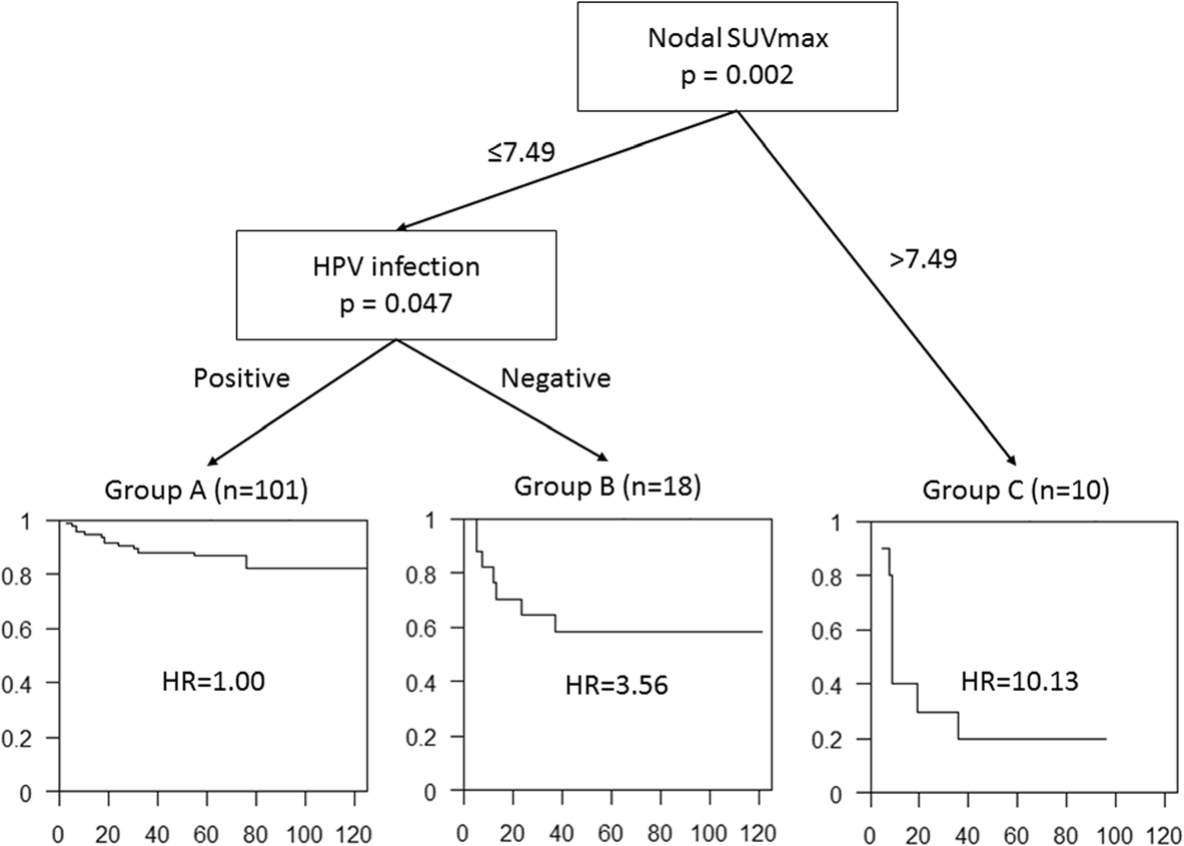
Classification and regression tree. Classification and regression decision tree (CART) analysis was performed to verify the prognostic factors. Square boxes indicate subsets of patients defined by the sequential splitting process. Finally, the CART analysis identified three risk groups: Group A (nodal SUVmax ≤7.49 and HPV positive), Group B (nodal SUVmax ≤7.49 and HPV negative), and Group C (nodal SUVmax > 7.49). Cox proportional hazard model calculated hazard ratios (HRs) of Group B (HR, 3.56; *p* = 0.006) and Group C (HR, 10.13; *p* < 0.001), compared to Group A (HR, 1.00)

**Fig. 2:**
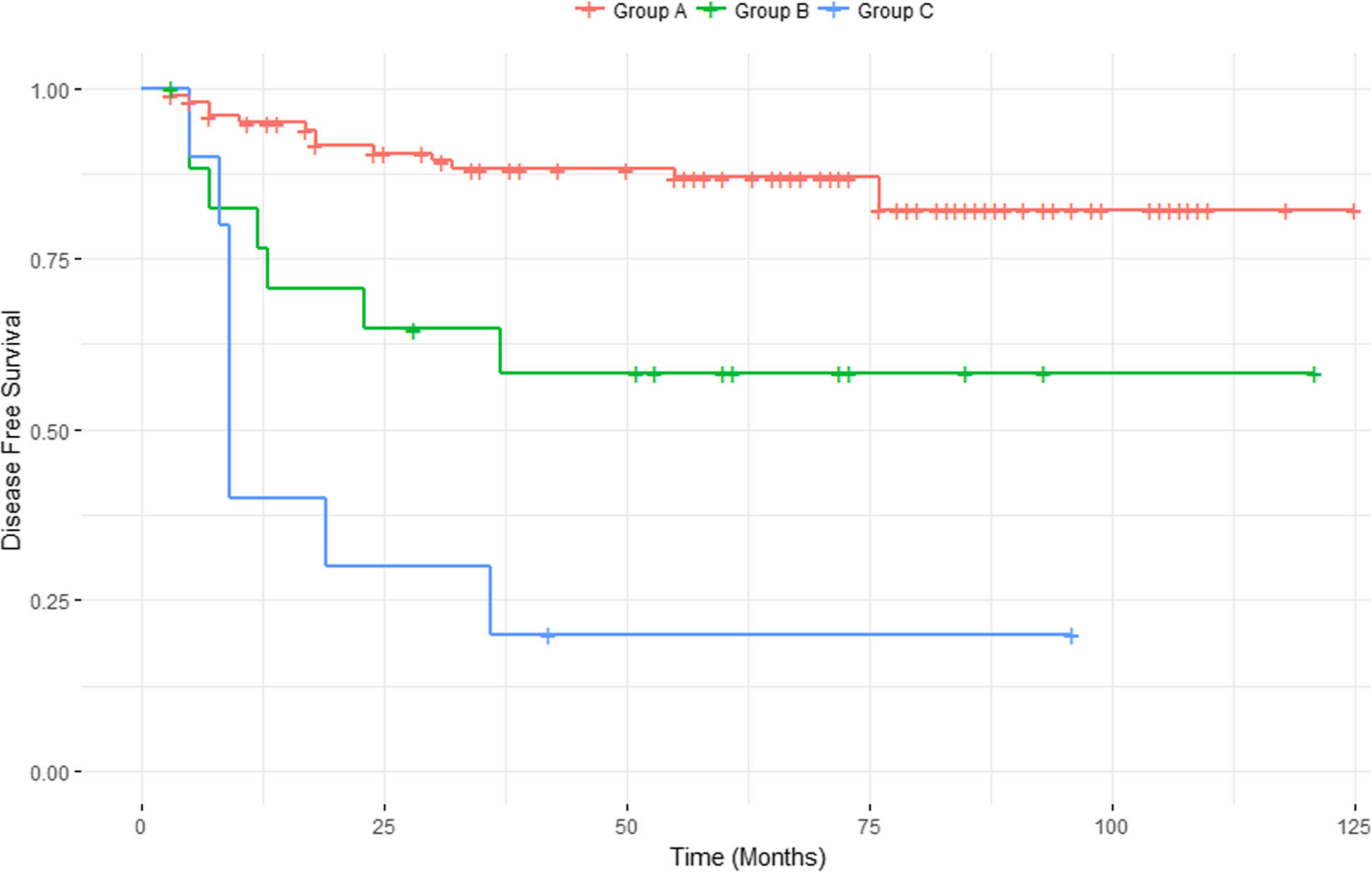
A Kaplan–Meier curve of disease-free survival. Classification and regression decision tree (CART) analysis showed three risk groups; Group A (nodal SUVmax ≤7.49 and HPV positive), Group B (nodal SUVmax ≤7.49 and HPV negative), and Group C (nodal SUVmax > 7.49). Log-rank test showed statistical significance among these groups (*p* < 0.001)

The CART analysis showed three risk groups based on nodal SUVmax and HPV status (Fig. 1): Group A (nodal SUVmax ≤7.49 and HPV positive), Group B (nodal SUVmax ≤7.49 and HPV negative), and Group C (nodal SUVmax > 7.49). There were 101 patients (78.3%) in Group A, 18 patients (14.0%) in Group B, and 10 patients (7.8%) in Group C (Fig. 1). Fourteen patients (13.9%) of Group A, seven patients (38.9%) of Group B, and eight patients (80.0%) of Group C showed recurrence during the follow-up period (Fig. 2). Cox proportional hazard model calculated HRs of Group B (HR, 3.56; CI, 1.44–8.85; *p* = 0.006) and Group C (HR, 10.13; CI, 4.17–24.57; *p* < 0.001), compared to Group A (HR, 1.00). DFS was significantly different among the three groups in the log-rank test (*p* < 0.001).

## Discussion

In this study, we investigated the prognostic variables, including traditional factors, such as age, FIGO stage, tumor size, lymph node metastasis status, and SCC antigen levels, as well as PET parameters and HPV status in cervical squamous cell carcinoma patients who were treated by CCRT. Tumor size, para-aortic lymph node metastasis status, nodal SUVmax, and HPV infection status were significant prognostic factors.

We established a risk stratification method, which can be performed simply using nodal SUVmax and HPV status. We used the CART analysis to evaluate the factors highly associated with DFS, which showed correlation in univariate analysis. The CART analysis provides the results which can be easily understood and interpreted. The CART analysis showed that nodal SUVmax is the most powerful significant prognostic factor and that HPV infection status is also a significant prognostic factor in patients with lower nodal SUVmax.

CART analysis is a simple yet powerful analytic tool that helps determine the most important (based on explanatory power) variables in a dataset [17]. This process is mathematically identical to certain familiar regression techniques; however, it has many advantages over more traditional methods, such as multivariate regression analysis [18]. The CART algorithm generates a decision tree, and it does not need to modify or classify the factors before analysis. Even continuous variables (such as, age and SUVmax) can be simply applied, and the algorithm generates the cut-off value to divide the patient population. The highest node separates the patient population into two groups, and then, each group can be further divided to subgroups. In other words, the most powerful prognostic factor is considered as highest node, and the second highest node means the most powerful prognostic factor in the subgroup. In this study, nodal SUVmax was the highest node in the CART analysis, and HPV infection status was the second highest node in patients with nodal SUVmax ≤7.49. However, patients with nodal SUVmax > 7.49 did not show significant factors to separate the patients in the CART analysis, which may be associated with there being relatively fewer patients in Group C.

In this study, nodal SUVmax showed powerful prognostic power in multivariate analysis, and it showed highest-order risk factor of DFS on the CART analysis. Previous studies also showed the prognostic significance of nodal SUVmax [6, 16]. Metabolic parameters from F-18 FDG PET/CT were widely studied to validate the prognostic values. Chong et al. compared prognostic values of pSUVmax, nodal SUVmax, metabolic tumor volume (MTV), total lesion glycolysis (TLG), and other clinical parameters. They showed that nodal SUVmax was only prognostic factor among these metabolic parameters [6]. Heterogeneity factor from F-18 FDG PET/CT also showed additive value for prognostic prediction, combined with nodal SUVmax and whole-body MTV [16]. In addition, nodal SUVmax and nodal MTV showed predictive value for the recurrence of cervical cancer in patients who reached metabolic complete response after CCRT [19].

However, some studies have reported opposite results. Guler et al. demonstrated that MTV and TLG were not independent prognostic markers; however, they did not analyze nodal SUVmax [20]. Crivellaro et al. reported that pSUVmax, MTV, and TLG of primary tumor did not show statistical difference [21]. Previous studies consistently showed that pSUVmax did not have prognostic predictive value on multivariate analysis, but the results for MTV have been discordant. One major reason is different methods in the measurement of the cut-off value to generate MTV in each study. Chong et al. and Son et al. considered the cut-off value as the mean SUV plus two standard deviations of the mediastinal blood pool [6, 16, 19]; Guler et al. considered the cut-off value as a fixed SUV value of 2.5 [20], and Crivellano et al. used automatic segmentation software for measuring MTV.

There are controversial reports about the prognostic values of SUVmax [6, 16, 22]. Primary tumor SUV represents not only tumor-associated inflammatory cells but also cervicitis and other combined inflammations. As F-18 FDG uptake of primary tumor is correlated with various factors, including the density of viable carcinoma cells, proliferation activity, angiogenesis, and tumor microenvironment [23, 24], we cannot differentiate FDG uptake from cancer and combined inflammation in current F-18 FDG PET/CT imaging. Therefore, previous data using metabolic parameters (pSUVmax, MTV, and TLG) of primary tumor might have shown discordant results [16]. However, nodal SUVmax represents tumor aggressiveness and is less affected by inflammation related to exogenous pathogens, and thus, it can be considered a more powerful prognostic marker.

Even though this study only evaluated pretreatment F-18 FDG PET/CT, recent studies have demonstrated that the metabolic responses on post-treatment F-18 FDG PET/CT could predict treatment outcomes in patients with cervical cancer [25, 26].

HPV infection status is a second-order risk factor in the CART analysis. HPV infection is a well-known etiology of cervical cancer, but some patients are HPV-negative. There are two possibilities of being HPV-negative in cervical cancer. One proposed mechanism of HPV-negative cervical cancer is transient HPV infection (a type of hit and run mechanism) [27]. As the virus is not essential for the maintenance of cancer, HPV virus is cleared by the host’s immunity after inducing cervical cancer [27]. However, a recent study based on The Cancer Genome Atlas (TCGA) cervical cancer database showed that HPV-negative cervical cancer shows difference in the molecular features (increased WNT/β-catenin signaling and somatic mutations in the TP53, ARID, WNT, and PI3K pathways) [28]. Therefore, we can assume that HPV-negative cervical cancer harbors a different etiology of cancer, and we need to approach the treatment of HPV-negative cervical cancer differently. Patients with HPV-negative cervical cancer show poorer prognosis compared to those with HPV-positive cervical cancer [8, 9], and our results also showed poor prognosis in HPV-negative patients. Interestingly, all Group C patients (nodal SUVmax > 7.49) were HPV-positive. Because of the relatively small number of the patients in Group C, further studies are needed to elucidate the relationship between nodal SUVmax and HPV status.

Previous studies have shown conflicting results with HPV infection subtypes [8, 14, 29–32], and prognostic values of each HPV genotype have not been elucidated. We analyzed prognostic values of HPV infection subtypes (Additional file 1: Table S2). Even though there were marginal survival predictive values of HPV 16 and alpha-9, there was no significant difference in this study. These conflicting data may be affected by differences in study designs, sample sizes, follow-up durations, and assay methods. As HPV negativity has more potent predictive value than HPV genotype analysis, we performed further analysis using HPV negativity.

Even though we performed the CART analysis including traditional risk factors, nodal SUVmax and HPV status were selected for classification. These two parameters are more potent predictors of prognosis in multivariate analysis and the CART analysis. FIGO staging system is widely used for the assessment of cervical cancer, but there are several limitations which can affect the optimal care of patients [33]. Previous reports showed that FIGO staging can be erroneous by 16–65% [33, 34], and FIGO stage does not reflect other risk factors, such as nodal status, HPV status, tumor volume, and metabolic parameters [4, 6, 8, 16].

Cervical squamous cell carcinoma patients with high nodal SUVmax or HPV negativity may need additional treatment after CCRT, such as targeted therapy or adjuvant chemotherapy. As patients with high nodal SUVmax showed poor prognosis to conventional CCRT, we can consider additional treatment to increase the survival of these patients. And additional targeted therapy may help the patients with HPV-negative cervical squamous cell carcinoma, which harbors molecular features that might better respond to new targeted therapy.

The present study had several limitations. This study was retrospective with a limited number of patients and there could be potential selection bias. As relatively old-generation PET/CT scanners were used in this study, SUVmax of modern scanners using advanced reconstruction methods could be different, particularly for small lesions. However, we tried to include a homogenous population of cervical squamous cell carcinoma patients who underwent CCRT as the initial treatment, and we performed daily and weekly quality control to maintain the PET/CT scanner following the guidelines of Korean Society of Nuclear Medicine. As external validation of the simple risk model, which is the result of this study, has not been conducted, future studies including larger population are needed for validating this model.

## Conclusion

The nodal SUVmax on pre-treatment F-18 FDG PET/CT and HPV infection status before CCRT are powerful, independent prognostic factors for the prediction of disease-free survival in patients with cervical squamous cell carcinoma who underwent initial CCRT. We also suggest a simple prognosis prediction model using pre-treatment FDG PET/CT and HPV genotyping, but it needs further validation in an independent dataset.

## Additional files

Additional file 1: **Table S1.** Univariate and multivariate analyses of disease-free survival. **Table S2.** Prognostic value of HPV infection subtypes. (DOCX 18 kb)

## Data Availability

The datasets used and/or analyzed during the current study are available from the corresponding author on reasonable request.

## Abbreviation

CART: Classification and regression decision tree
CCRT: Concurrent chemoradiotherapy
CI: Confidence intervals
DFS: Disease-free survival
DNA: Deoxyribonucleic acid
EBRT: External beam radiotherapy
FDG: F-18 fluorodeoxyglucose
FIGO: International Federation of Gynecology and Obstetrics
HPV: Human papillomavirus
HR: Hazard ratio
MTV: Metabolic tumor volume
nSUVmax: SUVmax of lymph nodes
PET/CT: Positron emission tomography/computed tomography
pSUVmax: SUVmax of the primary tumor
SUVmax: Maximum standardized uptake
TLG: Total lesion glycolysis
