# Supplementary material for: Enhancing prognosis prediction using pre-treatment nodal SUVmax and HPV status in cervical squamous cell carcinoma
Source: Cancer Imaging. 2019 Jun 24;19:43. doi: 10.1186/s40644-019-0226-4 (PMC6591806; doi:10.1186/s40644-019-0226-4)
Supplement: Supplementary file 1 — Table S1. Univariate and multivariate analyses of disease-free survival. Table S2. Prognostic value of HPV infection subtypes. (DOCX 18 kb) [file 40644_2019_226_MOESM1_ESM.docx]

Table S1. Univariate and multivariate analyses of disease-free survival

| Variables | Univariate analysis | | | Multivariate analysis | | |
| --- | --- | --- | --- | --- | --- | --- |
|  | HR | 95% CI | p-value | HR | 95% CI | p-value |
| FIGO stage | 2.93 | 0.89–9.64 | 0.077 |  |  |  |
| Tumor size > 4.4 | 3.67 | 1.62–8.30 | 0.002 | 2.44 | 1.02–5.81 | 0.044 |
| Pelvic lymph node | 1.89 | 0.90–3.96 | 0.094 |  |  |  |
| Para-aortic lymph node | 4.21 | 1.89–9.40 | <0.001 |  |  |  |
| Nodal SUVmax > 7.49 | 7.63 | 3.32–17.52 | <0.001 | 6.79 | 2.31–20.00 | <0.001 |
| HPV negative | 2.37 | 1.01–5.55 | 0.047 | 4.53 | 1.71–12.02 | 0.002 |

HR, hazard ratio; FIGO, International Federation of Gynecology and Obstetrics; SUVmax, maximum standardized uptake; pSUVmax, SUVmax of primary tumor; nodal SUVmax, SUVmax of the lymph node with the highest FDG uptake; HPV, human papilloma virus

Table S2. Prognostic value of HPV infection subtypes

| Variables | Univariate analysis | | |
| --- | --- | --- | --- |
|  | HR | 95% CI | p-value |
| HPV 16 | 0.78 | 0.28–1.21 | 0.145 |
| HPV 18 | 0.42 | 0.06–3.08 | 0.393 |
| HPV 33 | 1.04 | 0.25–4.37 | 0.959 |
| HPV alpha-7 | 0.92 | 0.22–3.87 | 0.908 |
| HPV alpha-9 | 0.51 | 0.24–1.07 | 0.076 |
